# Can Reclaimed Water Be Used for Sustainable Food Production in Aquaponics?

**DOI:** 10.3389/fpls.2021.669984

**Published:** 2021-06-04

**Authors:** Liliana Cifuentes-Torres, Gabriel Correa-Reyes, Leopoldo G. Mendoza-Espinosa

**Affiliations:** Oceanographic Research Institute, Autonomous University of Baja California, Ensenada, Mexico

**Keywords:** integrated farms, wastewater reuse, sustainability, reclaimed water, aquaculture, ornamental fish

## Abstract

Aquaculture is a technology used for the production of animal protein but produces a great amount of waste that decreases productivity and adversely affects the environment. Sedimentation and filtration have been used for the treatment of the suspended fraction of these wastes although dissolved substances like nutrients can be an asset. Therefore, the management of aquaculture waste remains a challenge. Aquaponics is a technology that can eliminate dissolved N and P from aquaculture systems as they serve as nutrients for plants, which are absorbed through the roots and are incorporated into their tissues. Several reports and studies exist on the benefits of aquaponic systems for the combined production of plants and aquatic organisms and its advantages in terms of economics and environmental protection. The great majority of the studies use the wastewater from the aquatic production tanks as a source of nutrients for plants production. However, domestic or municipal wastewater is a resource that has been used extensively in other production systems such as conventional agriculture and aquaculture, yet its potential as a source of water for aquaponics has not been established. The current analysis hypothesizes that reclaimed water can be used for aquaponics. Despite the extensive use of reclaimed water in agriculture and aquaculture and the low risk to human health when properly managed, there are no academic studies that have tackled this issue. In order to overcome the generalized mistrust of the public in consuming crops irrigated with reclaimed water or fish growing in reclaimed water, it is recommended that only ornamental fish and plants would be cultivated by this method. There is an urgent need for studies to verify the safety and advantages of such cultivation technique. Finally, it is necessary to establish guidelines for the responsible use of reclaimed water in aquaponics.

## Introduction

One of the challenges that science currently faces is the optimization of traditional production systems in ecological, economic, and social terms. The production of food in a sustainable and safe manner requires the recycling and improvement of ancient techniques like hydroponics which, since Babylonia times, already produced quality crops and provided urban landscaping (González-Carmona and Torres-Valladares, 2014). Currently, hydroponics is used as a technique that facilitates the growth of crops in cities, thanks to the implementation of urban farms and vertical gardens (Al-Chalabi, 2015). In these places another technique for food production has been implemented, in which in a synergetic way, hydroponics is integrated with waste generated by aquatic organisms in an aquatic recirculation system (Goddek et al., 2015). This type of production system is known as aquaponics and takes advantage of the symbiotic relationship between aquatic organisms, plants and bacteria (Rakocy, 2012). In aquaponics, two products are obtained that can be either edible or of economic value and that can be commercialized. Therefore, the combination of cultivation systems for fishes and aquatic plants such as duckweed and water hyacinths is not considered aquaponics for the present analysis.

Several studies have highlighted the potential of aquaponic systems (Diver, 2006; Endut et al., 2011; Rakocy, 2012). In all of them, aquaponics has been used as a treatment system for wastewater from aquatic systems. However, there are no studies in which reclaimed water has been used in such systems. The nutritional potential of reclaimed water has been identified in many studies in developed countries (Bixio et al., 2008), and public policy regarding the use of this type of water generally requires advanced treatment and disinfection (United States Environmental Protection Agency, 2012).

In developing countries, the treatment of wastewater is not common. It has been estimated that approximately 80% of the wastewater generated worldwide is discharged without proper treatment (Winpenny et al., 2013). Regardless of this, the FAO has promoted the concept of integrated farming in which all available resources, both agricultural and livestock, are used in a sustainable way, contributing to increase the quality of life of farmers and improving the natural environment. Some of such practices established by the FAO is the recycling of nutrients from livestock in fish cultivation such as tilapia. Therefore, it can be asserted that potentially, reclaimed water can be used after an adequate treatment for the cultivation of plants and fish, therefore, aquaponics.

In the present analysis, wastewater is referred to as crude wastewater that has not received any type of treatment, while reclaimed water is wastewater that has received some sort of treatment, usually secondary treatment. Taking this into account, our hypothesis establishes that it is possible to integrate in a planned and responsible way the use of reclaimed water as a source of nutrients in aquaponics systems, reducing the need to add synthetic nutrients to the system, thus, making the production more environmentally friendly. This last issue is important from a commercial point of view due to the fact that organic products usually have a higher value than non-organic ones, that results in higher income for the producer, and a shorter return in investment. The present article will analyze traditional aquaponics and highlight the potential for the use of reclaimed water in aquaponics and the challenges that lay ahead.

## Traditional Aquaponics

Aquaponic systems consist of a unit that cultivates plants without soil and another with a tank cultivating fish. The excreta from the fish are used as nutrients by the plants (Maucieri et al., 2018; Yep and Zheng, 2019). This type of production system was called integrated system (Rakocy, 2012), and has its origin is sustainable agriculture, that has as its goal to achieve the production of plants and livestock by using efficiently the resources, without damaging the environment by integrating the natural cycles in production systems to increase the quality of life of farmers and the society as a whole (The Food Agriculture Conservation and Trade Act of 1990, 1990). Therefore, aquaponics can be considered a sustainable agricultural system.

Some of the advantages of aquaponics systems are the efficient use of water (95–99%), the lower need for the addition of synthetic fertilizers (<50%), the elimination of the need of agrochemicals for pests and diseases control, the non-dependence to soil, the simultaneous production of plants and aquatic organisms and the low discharge of waste to the environment (Al-Fedh et al., 2008; Lampreia, 2016; König et al., 2018; Maucieri et al., 2018).

Historically, the species of fishes that have been commonly used in aquaponics are, in order of its frequency of use: tilapia (*Oreochromis niloticus*), catfish (*Clarias gariepinus*), carp (*Cyprinus carpio*), trout (*Oncorhynchus* mykiss), and pacu (*Piaractus mesopotamicus*) (Rakocy, 2012; Love et al., 2014a). Ru et al. (2017) highlights that due to its high tolerance to suspended solids, levels of nitrite above 44.67 mg L^–1^ and low concentrations of oxygen, *O. niloticus* is the most common species cultivated in commercial systems. With respect to the hydroponic component, the most common type of crops cultivated are leafy vegetables due to their ability to grow at high N concentrations, their shorter growth period, their relatively low nutrients requirements and their high demand (Bailey and Ferrarezi, 2017). Additionally, Love et al. (2015) found that the most common species of crops in aquaponic systems are basil (*Ocimum basilicum*), tomato (*Solanum lycopersicum*), lettuce (*Lactuca sativa*), cabbage (*Brassica oleracea*), beetroot (*Beta vulgaris*), pak choi (*Brassica campestris*), peppers (*Capsicum annuum*), and cucumber (*Cucumis sativus*). Exotic species of plants such as *Salicornia persica* have also been cultivated in aquaponic systems using brackish water. This has opened new windows into the possibility of using aquaponics as an option for the cultivation of marine organisms and plants tolerant to salt. Moreover, *S. persica* has a high concentration of lipids, omega 3 and minerals, making it attractive for cosmopolitan markets such as the European (Turcios and Papenbrock, 2014).

In a study by Hu et al. (2015) the dynamics of the nitrogen compounds in aquaponics systems with tomato (*Lycopersicon esculentum*) – pak choi (*Brassica campestris* L. *subsp. Chinensis*) – tilapia (*Oreochromis niloticus* was tested. Such study demonstrated that the assimilation of N varies with the type of crop, reaching 41.3% for tomato and 34.4% for pak choi. Such a difference is explained by the larger surface area of the roots of the tomato plant, that increases the amount of biofilm of nitrification bacteria, responsible for the oxidation of ammonia (NH_4_^+^) to nitrates (NO_3_^–^) (Endut et al., 2016). A lower concentration of ammonia was reached in water from tomato than from pak choi. The importance of the surface area of the roots for the removal of nutrients was mentioned by Endut et al. (2016). They used an aquaponics system with water spinach (*Ipomoea aquatica*) – green mustard (*Brassica juncea*) – catfish (*C. gariepinus*) and found that the removal of N compounds and orthophosphates were very similar but was more efficient for water spinach than for green mustard. Spinach had roots with larger surface area than green mustard and nutrients removal was 88.76 vs. 78.21% for ammonia, 92.51 vs. 86.67% for nitrite, 90.04 vs. 86.87% for nitrate, and 88.99 vs. 78.72% for orthophosphates. Henfi et al. (2015) evaluated the decrease of nutrients in aquaponic systems of *Cherax quadricarinatus* – *I. aquatica*. The rate of survival of *C. quadricarinatus* was 90% while the removal of nutrients was 84.6, 34.8, and 44.4% for NH_3_, NO_3_^–^ and orthophosphates.

Some factors that affect the dynamics of N in aquaponic systems are pH, dissolved oxygen, the hydraulic loading rate and the C:N ratio. The pH affects all organisms that interact in the aquaponic system. In the case of nitrification bacteria, it has been demonstrated that their activity decreases when pH is below 6.4 and greater than 9.0, and NUE (Nitrogen Use Efficiency) is 50.9% at pH 6.4 (Ruiz et al., 2003; Zou et al., 2016). This value is relatively high as it has been demonstrated that one of the problems with aquaponics is its low NUE (40%) (Hu et al., 2012; Wongkiew et al., 2017a) Dissolved oxygen (DO) also has a direct effect on nitrification bacteria. In the case of ammonia oxidizing bacteria (AOB), their transformation efficiency decreases at DO levels below 4.0 mg l^–1^ while at less than 2 mg L^–1^ the activity of nitrite oxidizing bacteria (NOB) is greatly reduced. Therefore, levels between 5 and 6 mg L^–1^ are recommended, which is ideal for the majority of aquatic organisms (Kim et al., 2005; Rakocy, 2012). The hydraulic loading rate (HRL, m^3^ day^–1^), which is the liquid flowrate per unit of cultivation area, is a variable that affects the retention time of nutrients, sediments and microorganisms (Li et al., 2009). A low HRL can cause a decrease of OD, while a high HRL can reduce the retention time of water, that can cause a decrease in the assimilation of nutrients by the crops’ roots and the washing of the bacterial biofilm thus, the deterioration of water quality (Endut et al., 2010). Finally, the C:N ratio is related with the population of nitrification bacteria and heterotrophic bacteria that coexist within the system. A high C:N ratio increases the growth rates of heterotrophic bacteria and decreases the level of nitrification bacteria (Ebeling et al., 2006). Also, a high population of heterotrophic bacteria decreases the concentration of DO and causes an inefficient transformation of ammonia to nitrate which can cause problems to the survival of fishes that can be exposed to toxic levels of NAT and nitrites (Wongkiew et al., 2017b).

The majority of the studies have demonstrated successful results in terms of nutrients recycling and assimilation. Buzby and Lin (2014) undertook a study in which the hydroponic element was isolated from the aquatic cultivation pond in order to evaluate nutrients uptake independently. The cultivation tanks with *L. sativa* and *Tropaeolum majus* were spiked with ammonia and water samples were taken every hour for 4 h. It was found that both types of plants were effective in reducing ammonia (81% with *L. sativa* and 89% with *T. majus*).

A study by Buzby and Lin (2014) demonstrated that it is unclear the relation between the amount of nutrients added to the aquatic culture and the removal efficiency by the hydroponic system; aspects like the species used for cultivation, the type of system, cultivation density and others have to be taken into consideration. Rakocy (2012), Al-Fedh et al. (2008), and Endut et al. (2010) studied this relation and obtained values between 14 and 42 g food m^–2^ to 60–100 g food m^–2^. Additionally, fish cultivation commonly requires the addition of synthetic fertilizers because normal fish food generally lacks nutrients such as K, Ca, and Fe (Graber and Junge, 2009).

Delaide et al. (2016) studied the effect of supplementing water with high-purity mineral salts for fishes in an aquaponics system to simulate a commercial hydroponic solution. They used three types of water: commercial hydroponic solution (HP), water for aquaponics non-supplemented (AP) and supplemented water for aquaponics (CAP). The study consisted in evaluating the nutrient concentration of leaves and the weight of sprouts and roots. Results showed that sprouts had a significantly higher weight with CAP compared to HP and AP. However, the weights obtained in HP and AP suggest that AP can be an alternative for conventional hydroponic systems. Results for roots showed that AP and CAP were larger than HP. The authors attributed this to the fact that water for the fish cultivation contained components like organic matter, rhizobacteria and fungi that stimulated the growth of roots and, in return, increased nutrients intake. Other substances in SRA that promote the growth of sprouts and roots are humic acids and phenols (Spaccini et al., 2009; Hambly et al., 2015).

In this respect, the addition of probiotics to the food for fishes in cultivation has been evaluated and it was found that the addition of microorganisms like *Saccharomyces cerevisiae*, *Lactobacillus acidophilus*, *Bacillus subtilis*, *Aspergillus oryzae*, *Rhodopseudomonas*, *Actinomyces*, and *Nitrobacter* have a significant effect in the feed conversion ratio (FCR), an increase in the production of catfish and the efficiency of organic matter removal in aquaponic systems (Zhou and Wang, 2014; Santoso and Sunadji, 2020). This is caused by the ability of probiotics to facilitate the absorption of nutrients in the digestive system of fishes by increasing the production of digestive enzymes like proteasas. An effect on the immune system of fishes has also been observed, due to the fact that the presence of beneficial bacteria suppresses the population of pathogenic microorganisms. All combine to a better growth of aquatic species in cultivation (Zhou and Wang, 2014).

Zahidah et al. (2018) studied the use of the Red Water System (RWS), a system that uses probiotics from the fermentation of *Lactobacillus casei* and *Saccharomyces cerevisiae*, for the cultivation of catfish. This technique uses probiotics for the decomposition of organic matter and the fact that probiotic microorganisms can reduce ammonia by oxidation in cultivation ponds. In aquaponic systems, the benefit is evident as the decrease of ammonia that cannot be undertaken by plants can be undertaken by these microorganisms. In an experiment by Zahidah et al. (2018) the concentration of ammonia decreased when the microbial activity in the RWS started, which allowed for the transformation of ammonia into nitrates. It was demonstrated that probiotic microorganisms are capable of degrading residual organic matter from food and feces, thus preventing its accumulation in aquaponic systems and improving the water quality of the culture.

Love et al. (2014b) evaluated the worldwide use of ornamental fishes and plants in aquaponic systems. They found that 48% of the cultivation systems had, as a primary organism, an ornamental fish like koi, goldfish or tropical fish. In contrast, only 20% of plants used in aquaponics were ornamental plants. A study by Mchunu et al. (2018) found that in South Africa, in 45 aquaponic systems located in main cities, 25% cultivated ornamental plants and 16% ornamental fish.

Aquaponic systems could be implemented in urban areas, effectively taking part in what is known as urban agriculture. The definition of urban agriculture includes the production, process and merchandising of foods within urban or peri-urban areas, through techniques like horticulture and aquaculture, optimizing the use of resources and improving the nutritional value of the products and creating jobs (Hernández, 2006; Ribeiro et al., 2015). In most cases, urban agriculture is undertaken in small scale systems and dispersed throughout the urban area. This type of agriculture has grown in popularity to supply food to a growing population established in urban settlements (Lampreia, 2016). This is key to provide a stable access to affordable food and avoid the so called nutritional desserts, that lead to nutritional and public health problems (Tomlinson, 2017). Thus, studies on urban aquaponics have grown in the last decade, and particularly after 2019, according to Wirza and Nazir (2020). Such studies have dealt with the social acceptance of urban aquaponics and its role in urban planification. Pollard et al. (2017) studied focal groups with experience in the production of urban foods, food distribution and business administration, by means of interviews and their opinion on urban aquaponics in the city of Adelaide (Australia). The majority of those interviewed were not familiar with aquaponics and there was a persistent negative opinion about the technology. This was attributed to the lack of knowledge and due overall fear to a more competitive new production technology. To solve this, the authors recommend to plan and create public policies to promote and facilitate the concept of aquaponics to the market, guaranteeing long-term profitability.

Having said that, aquaponics does present challenges. Rakocy (2012), Goddek et al. (2015), and Greenfeld et al. (2019) evaluated the profitability of aquaponic systems and concluded that its feasibility is complex due to several factors like the type of system used, the type of organisms in cultivation, the location and the size of the production. Likewise, Vermeulen and Kamstra (2013) found that aquaponics appears to be a production system less efficient than sustainable practices in organic agriculture, because factors such the recirculation of nutrients, energy efficiency and the optimization of land tend to be more expensive than traditional methods. However, the optimum ratio between the cultivated organisms and the development of less expensive technological systems for water treatment makes the technology more profitable (Goddek et al., 2015; Greenfeld et al., 2019).

In relation to public policies on urban aquaponics, the European Union has begun to plan the integration of policies and strategies of areas such as agriculture, fisheries, food production and environment and recognize the importance of aquaponics in each of these fields. The goal of these policies are to promote innovation, increase competitiveness, increase sustainability, increase the quality of the resources, optimize the use of land, contribute toward the wellness of organisms being cultivated and achieve economies with lower carbon footprint. The EU program is designed to provide financial backing for research projects and commercial assistance to people and businesses interested in establishing a business in aquaponics (Hoevenaars et al., 2018).

It is clear, thus, that aquaponic systems are an alternative for the production of food and the improvement of water quality. The symbiotic relations found in such systems, promotes the growth of bacterial communities and fungi that favors the growth of roots and increases nutrients assimilation. However, to date the optimum relationship between aquatic organisms and plants is not clear, and factors such as species, age and feeding habits influence the system so a “standard” value for its success is not yet possible. More investigations on these factors and the interaction of these factors is needed.

## Use of Wastewater in Agriculture and Hydroponics

Taking into consideration that approximately 57% of the energy used in agriculture is used for the production of nitrogen fertilizers (Yep and Zheng, 2019), and that it has been calculated that the reserves of phosphate will decrease down to half in the following 60 years, meaning that the cost of extraction will increase significantly (Goddek et al., 2015), it is of great importance to have new sources of nutrients. Reclaimed water could be such a source.

The increase in human population has generated an increase in the demand of food and greater pressure on natural resources such as water. This has led to countries such as Australia, United States of America, China, Israel, and Spain to include in their water management policies the reuse of reclaimed water for the irrigation of crops (Pescod, 1992). The success in its implementation with the production of fruits and vegetables has modified the water-food nexus in countries with arid and semiarid climate, and has established the reuse of reclaimed water as a viable water management option (Elgallal et al., 2016). Many studies have been published on the use of wastewater for agriculture (Gupta et al., 2010; Licciardello et al., 2018; Salgot and Folch, 2018) while its use in hydroponics has been demonstrated in lab-scale and pilot-scale experiments but examples of full scale commercial systems are very limited (Cifuentes-Torres et al., 2020).

## Use of Wastewater in Aquaculture

Animal protein and other products of aquatic origin can be supplied through aquaculture; this sector has reported an annual growth of 5.3% from 2001 to 2018 (Food and Agriculture Organization of the United, 2018) yet it has been estimated that approximately 75% of the nutrients are not used by the cultivated organisms, which results in environmental pollution and other aquaculture systems downstream (Liu et al., 2021). The use of reclaimed water can be a sustainable and dependable alternative for aquaculture. For example, reclaimed water can reduce the geographical dependency of aquaculture to freshwater, so that it can be located anywhere near a city with wastewater treatment plants. This would result in a decrease in production costs and the procurement of fresh produce of high protein content to the benefit of local communities (Zaibel et al., 2020).

The first experiences with the cultivation of fish using wastewater were in Germany at the end of the XIX century, in which trout, carp and salmon were cultivated in tanks fed with sewage-field drains (Prein, 1990). In China and India wastewater with high levels of nutrients, are traditionally considered an input for aquaculture because they promote the growth of plankton and other microorganisms that are food for fishes. In India, for example, the yield reached in ponds with reclaimed water cultivating tilapia and carp was 5 t ha^–1^, which represents 16% of the total sales of carp and tilapia in the municipality of Calcuta (Adhikari et al., 2009). Vo and Edwards (2005) reported a production of 7 t ha^–1^ of Tilapia Mozambique (*Oreochromis mossambicus*), Tilapia nilótica (*O. niloticus*), two Indian major carps (rohu, *Labeo rohita* and mrigal, *Cirrhinus mrigala*) and Chinese silver carp (*Hypophthalmichthys molitrix*) in 330 ha of ponds cultivating fishes in periurban areas in Vietnam.

Nowadays, there are numerous studies dealing with the risks of using reclaimed water for crops irrigation or aquatic organisms and take into account the high probability of microbial pollution, toxic metals accumulation, and emerging contaminants. Terechovs et al. (2019) evaluated the effect of 49 emerging contaminants in reclaimed water in fishes of the species *Bidyanus bidyanus* in the semi-rural region of Shoalhaven in Australia. They detected 20 emerging contaminants in the reclaimed water and 23 and 19 emerging contaminants in flesh and liver of the fishes, respectively. However, the concentration of all contaminants was below the limit established by Australian authorities, with the exception of benzotriazol with a concentration of 675 ng L^–1^ in reclaimed water, well above the 7 ng L^–1^ established by the Australian legislation.

The use of reclaimed water in aquaculture interconnected with the production of crops was evaluated in Bangladesh by a company called “Agricuatics,” in the city of Mirzapur, with a population of 3,000–4,000 habitants and a wastewater production of 100 L s^–1^ (Drechsel and Hanjra, 2015). The system consisted of 5 tanks. The first tank received the effluent from the city’s wastewater treatment plant and worked as a sedimentation tank; the second tank one was designed to have a high hydraulic retention time and was sown with duckweed. The 3rd, 4th, and 5th tanks were used for the cultivation of fish (carp and tilapia) and its effluent was used for the irrigation of fruit trees. The production of fish was 15 t ha^–1^ y^–1^ and for duckweed was 220–400 t ha^–1^ y^–1^. All of the products (the fish and the fruits) were sold in the local market so the production system was self-sufficient.

In a study by Terechovs et al. (2019), 17β estradiol, diazepam, verapamil and trimethoprim were found in the liver of fish, which indicates that they can accumulate and be metabolized by this organ. Adhikari et al. (2009) studied the concentration of Pb, Cd, Cr, Cu, and Zn in water, sediments and internal organs of fish cultivated in ponds with wastewater from Calcuta (India). They found that Pb exceeded the maximum level allowed by the Canadian Environmental Quality Guidelines (22 μg l^–1^ vs. 7 μg l^–1^). The five toxic metals were detected in sediments although only Cd and Pb exceeded the maximum levels established by the EPA (Cd: 10.1 μg g^–1^ dw vs. 1.2 μg g^–1^ dw; Pb: 50.5 μg g^–1^ dw vs. 46.7 μg g^–1^ dw). The only toxic metal that appeared to be bioaccumulated by the aquatic organisms was Zn, mainly in kidneys. The concentrations of all five toxic metals in flesh were many times lower than the safety margins established by the WHO and FAO thus, fish were considered safe for human consumption.

A study by Mark et al. (2019), found that fish of the species *Clarias gariepinus* cultivated in ponds with domestic reclaimed water can have Fe and Cd levels within the maximum levels allowed by the FAO, WHO, and NOAA. The tissues that presented a higher bioaccumulation of toxic metals were the gills and liver with concentrations of 0.1 – 2.0 mg kg^–1^, which were still below the safety threshold for human consumption (1 – 2 mg kg^–1^). Although *Escherichia coli* was detected in levels of 104 UFC 100 ml^–1^ in reclaimed water and sediments of the cultivation ponds, the fish tissues did not present *E. coli* nor other pathogens like Salmonella and helminths. However, as with agricultural produce, it is necessary to use adequate hygiene measures such as proper wash and adequate cooking of the fish after harvest, disinfection of hands and appliances to avoid contamination by pathogens.

Sharma and Olah (1986), Sahoo and Singh (2015), and Li et al. (2017) documented the use of waste from a porcine farm for tilapia cultivation obtaining good results in terms of growth without detrimental effects of the cultivated organism nor human health concerns. Thus, it has been put forward that an adequate management of livestock wastes can decrease the probability of contracting diseases by virus and bacteria such as *Escherichia coli* or *Toxoplasma gondii* (FAO/WHO, 2014).

With the goal to prevent infections by bacteria and contamination of fish meat, the WHO established a maximum levels of certain microbiological parameters in water, for example 1,000 fecal coliform bacteria per 100 mL in water. And to avoid the risk of infection by helminths, water has to be free of helminth eggs to prevent diseases such as schistosomiasis, fasciolopsiasis and clonorchiasis (Blumenthal et al., 2000). The same authors also mention the need to monitor the microbiological quality of fish once they have been fished. The handling of the fish is particularly important since the concentration of bacteria can be particularly higher in guts than in muscle, so during the process of evisceration the risk of contamination to other parts of the fish is very high.

As mentioned before, toxic metals such as arsenic, cadmium, lead and mercury can be bioaccumulated in carnivore fish; however, it is highly unlikely that the fish would be harvested at an adult age so the concentration of these metals would be low (WHO, 2006). The plants can also bioaccumulate toxic metals but at a level that is not considered a hazard to human health (WHO, 2008). In relation to chemical contaminants such as pesticides, these are, generally speaking, not a problem in the aquaculture industry. However, in countries with weak regulations and there is widespread use of agrochemicals, the possibility to be exposed to these substances increases substantially. Such is the case of glyphosate in waters of the Amazon region where it has been found bioaccumulated in tissues of fish for human consumption (Gómez-Ramírez et al., 2012).

The European Union (Unión Europea [UE], 2019) and Codex Alimentarius (Inter-Organization Programme for the Sound Management of Chemicals [IOMC], 2008) have established maximum levels of toxic metals allowed for in fish tissue for human consumption. For example, for Hg the maximum concentration in fish is 0.5 mg kg^–1^ fresh tissue; for Cd, it shouldn’t be above 0.050 mg kg^–1^ fresh tissue per day (Unión Europea [UE], 2019). For Pb, concentrations above 0.30 mg kg^–1^ can be considered a health risk and, according to The Joint FAO/WHO Expert Committee on Food Additives (JECFA), the weekly intake of inorganic As is 0.015 mg kg^–1^ (WHO, 2008).

Therefore, in order to avoid the risk for the consumers of fish cultivated with reclaimed water due to the bioaccumulation of toxic metals and microcontaminants, the cultivation of ornamental fishes can be an attractive option. It has been estimated that approximately 4,000 species of freshwater ornamental fish are commercialized worldwide, of which between 700 and 800 are cultivated. In contrast, only 180 species of freshwater fish are used as food (Ramirez et al., 2010). For ornamental fishes, wastewater of lower quality can be used, which means fewer wastewater treatment processes and higher profitability.

As highlighted above, more studies on the bioaccumulation of substances, using different species of fishes and changing cultivation conditions in order to gather further experiences with the cultivation of fish in reclaimed water are needed.

## Aquaponics With Reclaimed Water

It was not possible to find an academic study that used wastewater or reclaimed water for aquaponics. The only apparent study that does mention wastewater for aquaponics is Rana et al. (2011) that used domestic wastewater at various dilutions for the growth of tomato (*Lycompersicum esculentum*). Although the title does mention aquaponics, there is no description of the aquatic organism used nor about its growth or survival rates. Having said that, there are a considerable number of studies in which wastewater has been used for the growth of fish, plants or vegetables which might indicate that such activities could be integrated (into aquaponics) and become viable taking into consideration factors such as health standards, concentration of contaminants in water and monitoring of certain contaminants in the flesh of plants and fishes to avoid human health risks.

Some examples of such studies are the following. Siqwepu et al. (2020) demonstrated that the addition of 30 mg kg^–1^ of ferrous sulfate (FeSO_4_) to the diet of fish (*C. gariepinus*) increased their hematologic profile and produced an effluent adequate in terms of Fe (0.16 mg L^–1^) for the growth of plants. Luo et al. (2020) analyzed the influence of the use of Selenium (Se) on the growth, ornamental features and health of the Koi carp (*Cyprinus carpio Koi)* and lettuce. In diets using 1.55 – 1.57 mg Se kg^–1^ it was observed a greater weight gain, a larger content of carotenoids and improved immunological capacity of the carp. In the hydroponic system, the lettuce did not show adverse effects with the addition of Se. It was concluded that Se is a microelement essential for animals and a cofactor in glutathione peroxidase (GSH-Px), which is an important antioxidant and eliminator of free radicals. The use of reclaimed water for the cultivation of fishes does not adversely affect their growth rate and survival rate, according to Zaibel et al. (2020). They conducted a study with *Cyprinus carpio* cultivated in solutions of 0, 50, and 100% municipal reclaimed water for 5 months. Similarly to other studies, the concentrations of toxic metals in the flesh of the fish were below the maximum levels established by the FAO. These results can make us conclude that reclaimed water can be safely used for the cultivation in aquaponics for some species of fish.

Although no specific reports on the use of reclaimed water in aquaponics were found, several published studies have dealt with the treatment of wastewater during the cultivation of aquatic organisms, mainly through phytoremediation by aquatic plants such as water spinach (*Ipomoea aquatica*) and duckweed (*Lemna minor*). These plants are added as a remediation component (Effendi et al., 2015) or as a diet supplement of fishes and other organisms (Pinandoyo et al., 2019). Studies by Effendi et al. (2015) demonstrate the nutrient removal capacity of an aquaponic system with crayfish (*Cherax quadricarinatus*) and water spinach, obtaining 85% reduction for NH_4_^+^, 34% for NO_3_^–^ and 44% for PO_4_. Endut et al. (2009) used water spinach to treat wastewater from an aquatic system cultivating African catfish at three hydraulic loading rates (HLR). Results demonstrated the removal of 65% of BOD, 83% of total suspended solids, 78% of ammonia and 89% of nitrites and a positive correlation between removal rates and hydraulic loading rate (HLR). All hydraulic loading rates (HLR) were efficient for the removal of nutrients and to maintain water quality conditions for the growth of fishes.

Other studies have demonstrated that small densities of fishes in reservoirs filled with reclaimed water can help regulate the growth of microalgae and undesirable vectors such as mosquitoes and snails (Terechovs et al., 2019). The growth of microalgae in aquaponic systems has been used to promote the improvement of water quality by increasing its buffer capacity, dissolved oxygen levels and the production of polyunsaturated fatty acids that can be added to the diet of the fish. The latter is very relevant as a common deficiency of aquaponics systems (and indeed aquaculture systems) is the need to add external substances like concentrates, which represent one of the highest costs in aquatic production systems (Addy et al., 2017).

As mentioned earlier, other options for the use of reclaimed water in aquaponic systems is the use of ornamental plants (*Dianthus, Chrysanthemum, Gerbera, Euphorbia, Anthurium, Alstromeria, Lilium, Rose*) and ornamental fishes (Table 1). The worldwide market for ornamental plants has been calculated in 60 billion dollars per year. The countries with the larger demand for flowers are Switzerland, Japan and the United States and the main producers are the European Union, United States, Japan, and Colombia (van Uffelen and de Groot, 2005). Reclaimed water could serve as an important source of nutrients for the cultivation of ornamental plants although risks for human health need to be taken into consideration (De Bon et al., 2010). The market for ornamental fish in 2010 was calculated at 10 billion dollars. The largest importers of ornamental fish are the United States, the European Union and Japan, while the main exporters are Belgium, The Netherlands, United States, Australia, Brazil, and Colombia. It has been calculated that more than half of the total commerce for wildlife are fishes, and only in the United States there are more than 160 million aquariums (Biondo and Burki, 2014). Therefore, reclaimed water could be used for the production of ornamental fishes and it wouldn’t affect its commercialization as these would not be used for human consumption.

**TABLE 1:** List of ornamental fishes that can be cultivated in captivity and with the potential of being used in aquaponics with reclaimed water.

**Specie**	**Care level**	**Diet**	**Max. Size (cm)**	**Sale price (USD)**
*Cyprinus carpio*	Easy	Omnivore	7.6	$17.99
*Carassius auratus*	Easy	Omnivore	20.3	$2.69 (3 – 5 pack)
*Pterophyllum scalare*	Easy	Omnivore	15.2	$4.99
*Danio rerio*	Easy	Omnivore	6.4	$70 (10 pack)
*Poecilia reticulata*	Easy	Omnivore	4.5	$14.99 (3 pack)
*Puntius tetrazona*	Easy	Omnivore	7.6	$11.99
*Gymnocorymbus* sp.	Moderate	Omnivore	6.4	$11.99
*Symphysodon* sp.	Moderate	Carnivore	20.3	$479.99 (3 pack)

*Source: https://www.liveaquaria.com/. The costs are for organisms in aquariums and presented only as a reference. Costs are subject to change and depend on availability, quality and quantity of organisms required for each system.*

In relation to legislation for the use of reclaimed water in aquaponics, there are no current norms. The United States, the European Union and the WHO have guidelines for the use of reclaimed water in agriculture, in order to guarantee low risk to human health (Cifuentes-Torres et al., 2020). With respect to the risks associated with the use of wastewater in aquaculture and, therefore, applicable to aquaponics, the WHO has established that the primary concern is the presence of pathogens, followed by the exposure to chemical substances. The adequate treatment of wastewater can significantly decrease the transmission of illnesses. It has been established that other measures such as adequate cooking of the food and hygiene facilities within the households can decrease the risks of contamination of bacteria like *E. coli*, *Vibrio Cholerae*, *Salmonella* spp. and *Shigella* spp., protozoa like *Giardia intestinalis* and *Entamoeba* spp., and virus like hepatitis A, hepatitis E, adenovirus and rotavirus. The risk of disease by helminths such as *Ascaris*, hookworms and *Taenia* spp. is higher for farmers and consumers of contaminated plants than for aquaculture workers and consumers of contaminated fish. Special considerations have to be taken with nematodes such as *Clonorchis, Opisthorchis* y *Fasciola* which can be transmitted by direct contact with contaminated water or if the infected plant or fish are eaten raw.

In relation to the possible consideration of aquaponic products as organic, according to the guidelines established by the National Organic Standards Boards (NOBS), the addition of synthetic materials and products from an industrial nature, limit the possibility of obtaining an organic certification in products from aquaponic systems (National Organic Standards Board [NOSB], 2016). In these, inorganic substrates are commonly used for the hydroponic component and pelleted food is used for aquatic organisms (Kledal et al., 2020). Additionally, according to the regulations by the EU (834/2007), for the production of organic horticulture, plants have to grow on soil, using the biological interactions generated in this ecosystem and, thus reducing the addition of agrochemicals to the soil. This has created problems due to desertification, monocultures, and the decrease of nutrients in agricultural soils so for traditional agriculture it is a challenge to grow organic products (Altieri, 2002). This has caused the need to implement new technologies, like hydroponics, that partially solves the problem of the lack of nutrients in soil. Nevertheless, organic horticulture prefers to pursue soil management rather than the adoption of a technology that dispenses with soil, causing that the certification of products from aquaponics, hydroponics or aquaculture will probably won’t happen in the near future, specifically in the UE (Kledal et al., 2020).

Despite this panorama, some private certification agencies in the United States, approved by the USDA, are generating organic certifications to vegetables produced by aquaponics under the argument established by the National Organic Standards Board [NOBS] in 2002 that defines organic production as “a production system that follows the agreements by law and regulations through the promotion of the natural cycle of the resources, through the integration of cultural, biological and structural components (referring to the assembly of the production system), promoting the ecological equilibrium and conservation of biodiversity” (National Organic Standards Board [NOSB], 2016). Thus, the methods used by hydroponics and aquaponics are legally entitled to be certified for the production of organic products as long as the producer can demonstrate the use of organic products guidelines (National Organic Standards Board [NOSB], 2010).

However, there is still controversy on the use of organic labels in crops produced from soil-less technologies (such as hydroponics and aquaponics) due to the opposition by soil-based farmers that argue that new labeling could cause confusion for consumers (Agricultural Marketing Service [AMS], 2016). The reality is that consumers of products from soil-less systems do not necessarily seek organic-product labels to consider these products more environmentally friendly than those from traditional agriculture (Kledal et al., 2020). Considering the fact that fertilizers in aquaponics originate through reclaimed water with nutrients from other systems, the organic certification would make even more sense, when following all health-safety protocols.

The development of new policies for the use of reclaimed water in aquaponics must include, following the proposal by Alcalde Sanz and Gawlik (2017), the development of operational procedures such as: implementation of a risk management evaluation team, characterization of the reclaimed water, the effluent and receiving waters and processes validation. This management framework is similar to the one used to regulate the reuse of reclaimed water in irrigation and aquifer recharge.

The data included in the present analysis are those found in academic publications. It is evident that, in theory, the potential for the use of reclaimed water in aquaponics is high. However, the potential is only theoretical so there is a need for studies that can translate the potential into reality by means of experimental demonstrations, and pilot-scale studies would be particularly useful.

## Conclusion

Reclaimed water can, in theory, be used in aquaponics as it has been used as a water source in agriculture irrigation and aquaculture for many decades. The current analysis highlights that there is an opportunity to use reclaimed water in aquaponics although there are still many questions that arise and more studies are needed to demonstrate that this technology is sustainable. There is the potential that toxic compounds such as certain toxic metals at low concentrations can function as food supplies in fish diets, under strict and controlled conditions. The presence of microalgae in aquaponic systems can make it an advantage as it acts as both a food producer and wastewater treatment process. It is necessary to develop guidelines for the use of wastewater in aquaponic systems. To do so, it is necessary to continue studies with aquatic organisms and plants with the ability to metabolize contaminants without the risk to human health. Studies on the effects of water quality and possible bioaccumulation of contaminants in fish and plant tissue would have to be undertaken to prove its eventual safety and facilitate its commercialization.

## Data Availability Statement

The original contributions presented in the study are included in the article/supplementary material, further inquiries can be directed to the corresponding author/s.

## Author Contributions

LC-T, GC-R, and LM-E contributed to the conception of the manuscript and contributed to manuscript revision, read, and approved the submitted version.

## Conflict of Interest

The authors declare that the research was conducted in the absence of any commercial or financial relationships that could be construed as a potential conflict of interest.
